# Transfer Learning With Simulated and Recorded Data Improves Predictions of Lateral Pinch Thumb-Tip Forces

**DOI:** 10.1109/TNSRE.2026.3709075

**Published:** 2026

**Authors:** Kalyn M. Kearney, Tamara Ordonez Diaz, Joel B. Harley, Jennifer A. Nichols

**Affiliations:** J. Crayton Pruitt Family Department of Biomedical Engineering, University of Florida, Gainesville, FL 32611 USA; J. Crayton Pruitt Family Department of Biomedical Engineering, University of Florida, Gainesville, FL 32611 USA; Department of Electrical and Computer Engineering, University of Florida, Gainesville, FL 32603 USA; J. Crayton Pruitt Family Department of Biomedical Engineering, University of Florida, Gainesville, FL 32611 USA

**Keywords:** Artificial neural networks, electromyography, lateral pinch, musculoskeletal simulations, transfer learning

## Abstract

Machine learning is a powerful tool for modeling complex movements. However, the high cost of acquiring biomechanical data often limits observations and therefore the generalizability of machine learning models. Meanwhile, there is untapped utility in presently published musculoskeletal simulations for informing these predictions. This work evaluated whether a transfer learning model trained on both musculoskeletal simulation and recorded data outperforms a direct learning model trained on recorded experimental data alone. For the transfer learning model, we leveraged a simulation dataset with 6,594 lateral pinch simulations to train a long short-term memory neural network to predict three-component thumb-tip forces from muscle activations. We concatenated this pre-trained neural network with a feedforward architecture and used recorded data (n = 12 subjects) to fine-tune the model. The feedforward architecture used the outputs of the long short-term memory neural network, as well as static subject-specific features (e.g., anthropometric measurements). We then trained a direct learning model with a similar architecture using just the recorded data. The transfer learning and direct learning models were parameterized via a random search and underwent five-fold cross-validation. To elucidate the impact of pre-training with simulations, we validated both neural networks and compared their test errors using a leave-out set (n = 3 subjects). The transfer learning model outperformed the direct learning model, as suggested by statistically significant lower absolute errors (p = 0.04). Our results suggest the viability of using musculoskeletal simulations to bolster machine learning models that can expand the utility of simulated datasets and potentially improve predictions of real-world biomechanics.

## I. Introduction

ACCURATE predictions of upper extremity biomechanics are indispensable for a breadth of applications. For example, these predictions can guide the design of ergonomic assessments [1], prosthetic devices [2], and human-robot interactions [3]. In clinical settings, these predictions can improve quality of life through data-driven rehabilitation strategies [4] and surgical decisions [5]. Continued improvements to upper extremity models serve to expand their application and accelerate improvements to individual lives.

Two primary approaches have been used to model the upper extremity: musculoskeletal simulations and machine learning. Musculoskeletal simulations use actuators (i.e., muscle-tendon units) to act on rigid links (i.e., bones). Leveraging knowledge of physics, controls, anatomy, and physiology, musculoskeletal simulations can elucidate the cause-and-effect relationships that describe human movement (e.g., [6], [7]). However, musculoskeletal models rely on assumptions surrounding musculoskeletal geometry, joint motion, and muscle-tendon dynamics. These assumptions cause uncertainty in simulated outcomes [8], hindering their utility. Machine learning models require high-fidelity data (typically recorded data) to predict or characterize biomechanical systems. When ample high-quality data are available, machine learning models can rapidly predict complex biomechanical behaviors, facilitating the adoption of these models into everything from myoelectric controllers [9] to web-based smartwatch applications [10].

Biomechanical data are costly to acquire, challenging the development of robust, complex machine learning models. Most prior works leveraged less than 200 recorded subjects to train and test their machine learning models [11], and many recent studies continue to rely on limited subject cohort sizes (e.g., [12], [13]). Such sample sizes can constrain the complexity and generalizability of machine learning models. Some works have instead trained machine learning models using simulated data, demonstrating that simulated datasets can encode meaningful biomechanical characteristics [14], [15]. However, methods to improve machine learning model generalizability to recorded data, especially for systems as complex as the upper extremity, are needed. Transfer learning, the process of training a machine learning model on one task (e.g., simulated data) and re-purposing this knowledge for another task (e.g., measured data) [16], has therefore emerged as a tool for biomechanical modeling [17], [18], [19]. Notably, Park et al. reported improved generalizability during the prediction of ankle motions using a simulation-to-real data transfer learning approach, as compared against a model that learned directly from the recorded dataset [20]. We also recently demonstrated the effectiveness of simulation-to-real transfer learning for predicting elbow flexion torques with fatigue onset; this approach reduced test root-mean-square-errors by nearly 25% relative to a model trained directly on recorded data [21]. Importantly, both these studies focused on relatively simple tasks. Thus, there is a need to understand the utility of a simulation-to-real data transfer learning framework for predictions involving complex, multi-joint tasks and muscle activations, which are among the most burdensome features to collect *in vivo*.

Our objective was to elucidate the extent to which transfer learning improves artificial neural network (ANN) predictions of complex upper extremity movements, as compared to a conventional direct learning approach. We modeled lateral pinch, a task that involves the combined, redundant effort of muscles acting about the wrist and thumb. Specifically, our ANNs predict time-series three-component thumb-tip forces from static, subject-specific features (e.g., anthropometrics) and time-series muscle activations. Our transfer learning framework used simulated data to pre-train an ANN and recorded data to fine-tune the model. We also trained a separate ANN on recorded data alone. We hypothesized that the ANN informed by musculoskeletal simulations and recorded data (i.e., transfer learning model) would outperform the ANN informed by recorded data alone (i.e., direct learning model). Our results demonstrate the utility of simulation-to-real transfer learning for the prediction of lateral pinch thumb-tip forces, informing the development of biomechanical machine learning models trained on limited datasets.

## II. Methods

**Fig. 1.**
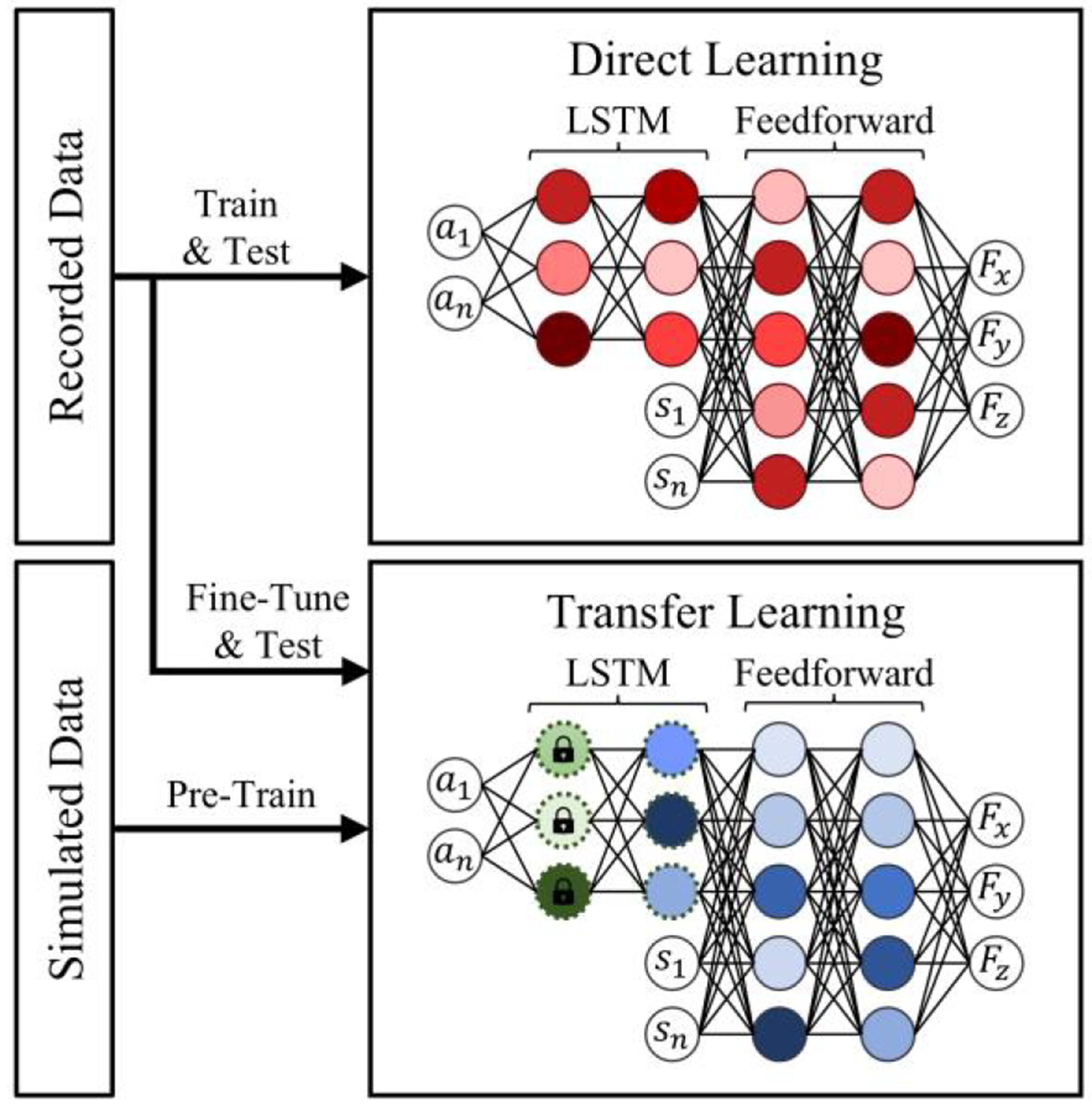
Overview of the direct learning (top) and transfer learning (bottom) models, which were defined as ANNs comprised of LSTM and feedforward layers (exact node and layer counts not depicted). Only recorded data was used in the direct learning model. Specifically, muscle activations (denoted *a*_1_ through *a_n_*) were inputs to LSTM layers. LSTM outputs and static subject-specific features (denoted *s*_1_ through *s_n_*) were inputs to feedforward layers. The outputs were thumb-tip force components, *F_x_*, *F_y_* and *F_z_*. In the transfer learning model, simulated muscle activations were used to pre-train the LSTM layers (represented by dashed outline). Then, the transfer learning model was fine-tuned using recorded data. During this fine-tuning, the hindmost layers of the pre-trained LSTM were kept unfrozen (blue nodes with dashed outline). Both the direct and transfer learning models were evaluated using a leave-out set, and the quality of their predictions were compared.

**Fig. 2.**
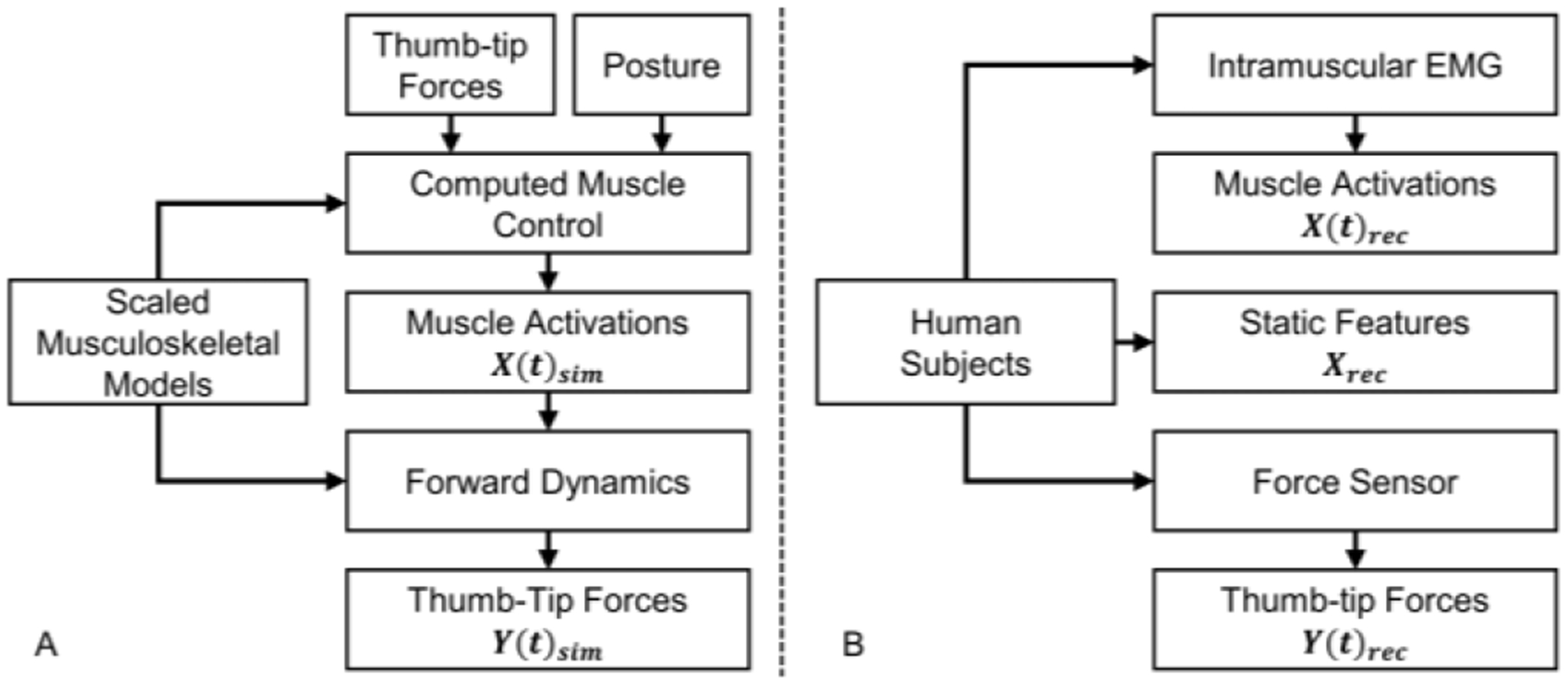
Pipelines for simulating **(A)** and recording **(B)** the lateral pinch datasets. Scaled wrist and thumb models were used in computed muscle control simulations that targeted a static posture and varying thumb-tip forces. The resulting muscle activations (*X*(*t*)_*sim*_) then served as inputs to forward dynamic simulations, from which thumb-tip forces (*y*(*t*)_*sim*_) were retrieved. We also recorded electromyographic signals (EMG) to acquire muscle activations (*X*(*t*)_*rec*_), as well as static subject-specific features (***X**_rec_*) and thumb-tip forces (*X*(*t*)_*rec*_). Muscle activations and static features were LSTM and feedforward neural network inputs, respectively. Thumb-tip forces were ANN outputs.

To validate our transfer learning framework for modeling lateral pinch, we developed two ANNs using PyTorch v. 2.0.1 [22]: a direct learning model and a transfer learning model (Fig. 1). To train and validate these ANNs, muscle activations and thumb-tip forces during lateral pinch were both computationally simulated and experimentally recorded (Fig. 2). As described in detail below, we used a generic musculoskeletal model of the wrist and thumb [23] in OpenSim v. 4.1 [24], [25] to simulate a large dataset representing lateral pinch for a healthy young adult population. We also recruited 29 healthy young adults as part of an IRB-approved study (University of Florida IRB #202202263). All provided written informed consent. Of those recruited, 15 subjects completed all recordings associated with lateral pinch trials. We developed direct learning and transfer learning models and performed leave-one-out cross-validation (LOOCV) using trials from 12 subjects in the training set. This effort informed whether transfer learning was consistently better (or worse) than direct learning across individual subjects. Using trials from the same 12 subjects, we tuned and trained the final direct learning and transfer learning models. Lastly, we used the final direct and transfer learning models to predict trials for the 3 leave-out subjects, and compared their errors.

### A. Simulated Dataset

**Fig. 3.**
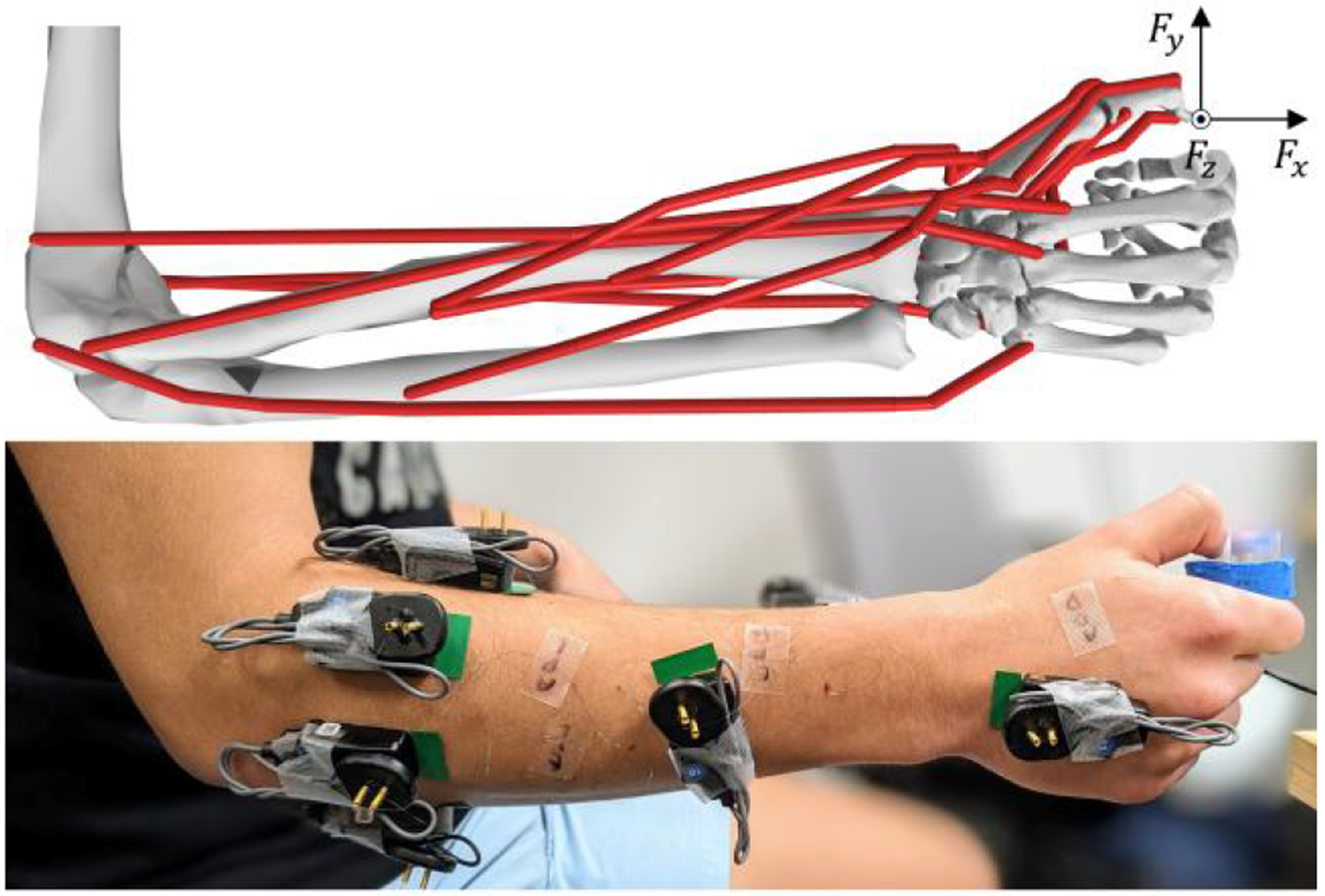
Generic musculoskeletal model used to simulate lateral pinch’ (top) and experimental setup used to record lateral pinch (bottom). *F_x_*, *F_y_*, and *F_z_* represent thumb-tip force components in the distal, dorsal, 4 and ulnar directions, respectively.

To provide observations to the transfer learning model, we employed simulation methods similar to Kearney et al. [14]. Specifically, we scaled a generic wrist and thumb model (Fig. 3, top) to represent 5th-95th percentile 20-29 year-old young adults based on known mass and height [26]. Each scaled musculoskeletal model was then input into multiple computed muscle control simulations. Computed muscle control uses proportional-derivative control and static optimization to determine the muscle activations required to achieve prescribed kinematic and force trajectories while minimizing muscle effort. These simulations targeted a static lateral pinch posture (−15 ° carpometacarpal flexion, −20° carpometacarpal abduction, 20° metacarpophalangeal flexion, 40° interphalangeal flexion) but varied the direction and magnitude of the target force. Specifically, target thumb-tip forces varied from 40 N to 80 N in 5 N increments. This target force range heavily overlaps with maximum lateral pinch forces reported for healthy adults [27]. Moreover, simulations with target forces exceeding 80 N frequently failed to converge. We applied these forces palmarly at the thumb-tip, as well as with 25% deviations in orientation in the distal, ulnar, and radial directions. These target forces were simulated across a linear 1.5 second ramp, with an initial force of 10 N, before plateauing at their maximum. Muscle activations from computed muscle control served as inputs to forward dynamic simulations, which solve the multibody equations of motion at each time step to predict musculoskeletal kinematics and forces. Thumb-tip forces were quantified at a point constraint on the distal phalanx of the thumb using joint reaction analysis, which resolved reaction forces into their distal, dorsal, and ulnar components. Using only those simulations that converged, the resulting dataset contained 6,594 simulations of lateral pinch representing 525 synthetically-generated, healthy young adults. In preparation for ANN training and testing, all simulations were interpolated to contain the same number of time instances, at approximately 100 Hz. The resulting simulated dataset contained only dynamic features (i.e., muscle activations and thumb-tip forces), which were used to pre-train the transfer learning model.

### B. Recorded Dataset

To train and test each ANN, we used the complete, recorded data of 15 healthy young adults (10 male, 5 female; 21.9 ± 2.6 years). These data included 8 static features: sex, height (1.74 ± 0.08 m), mass (76.2 ± 14.4 kg), maximum lateral pinch force magnitude (81.5 ± 19.1 N), hand length (18.4 ± 1.3 cm), hand width (8.9 ± 0.6 cm), forearm length (26.3 ± 1.6 cm), and forearm width (9.7 ± 1.0 cm). We used ultrasound to guide the insertion of fine-wire electrodes, which recorded electro-myography (EMG) from 8 muscles: *extensor pollicis brevis* (EPB), *extensor pollicis longus* (EPL), *flexor pollicis longus* (FPL), *abductor pollicis longus* (APL), *adductor pollicis* (ADD), *flexor carpi ulnaris* (FCU), *flexor pollicis brevis* (FPB), and the *opponens pollicis* (OPP). All EMG signals were recorded at 3000 Hz. We also used a six-axis force sensor to record three-component thumb-tip forces during lateral pinch (Fig. 3, bottom).

Each subject performed two tasks: lateral pinch maximum voluntary contractions (MVCs) and muscle-specific MVCs. First, each subject performed three MVCs of lateral pinch, as quantified by their thumb-tip force. Each MVC was performed across 5 seconds with 30 seconds of rest between each. During these trials, subjects were asked to maintain a static posture: neutral shoulder, 90° elbow flexion, neutral wrist, and thumb-tip centered on the force sensor. To avoid external forces not accounted for in the musculoskeletal simulations, subjects were instructed not to lean on any surfaces during each trial. Verbal encouragement and visual feedback of their thumb-tip force traced over time were provided during each trial. After the lateral pinch MVCs, each subject performed 8 muscle-specific MVCs (one per muscle recorded) as described by Kendall et al. [28]. During these trials, subjects received verbal encouragement and visual feedback of their EMG signal traced over time for the activated muscle.

The recorded lateral pinch muscle activations and thumb-tip forces were prepared for ANN training and testing. For each subject, the recorded thumb-tip forces were transformed to match the axes defined in the wrist and thumb model, and each trial was interpolated to the same length as the musculoskeletal simulations. The recorded EMG signal for each muscle was centered about zero and filtered with a Butterworth bandpass filter using cutoff frequencies of 20 Hz and 500 Hz. The signal was then rectified and filtered with a Butterworth lowpass filter using a cutoff frequency of 5 Hz. We then used a median filter with a window size of 2999 (i.e., approximately 1 second of data) to remove any remaining spikes. We scaled these data to a range of [0, 1] using the peak muscle activation observed over all trials (whether from lateral pinch MVCs or muscle-specific MVCs) for the subject. We then interpolated each lateral pinch trial to the same length as the musculoskeletal simulations. Lastly, all data were formatted together and split into training and test sets. Specifically, the data for 3 randomly-selected subjects (2 male, 1 female; ages 19, 23, and 27 years) were set aside for testing each ANN, while the remaining data were used for training. All anthropometric measurements associated with subjects in the test set fell within ±2 standard deviations of the training set means.

### C. Direct Learning Model

We developed an ANN trained directly on the recorded data, which predicted dynamic three-component thumb-tip forces from a combination of static (e.g., mass and height) and dynamic (i.e., EMG signals) features (Fig. 1, top). EMG signals were inputs to a long short-term memory (LSTM) neural network [29]. LSTMs are a type of recurrent neural network capable of recognizing patterns in time-series data, such as those present in EMG signals. This LSTM was then concatenated with a feedforward architecture, which received the outputs of the LSTM and each of the 8 static features. Static features were included to account for inter-subject differences that influence lateral pinch strength (e.g., anthropometric measures [30]). The ANN used an Adam optimizer, a mean-square-error (MSE) loss function, and applied weight decay to weights and biases to help prevent overfitting [31]. The ANN’s architecture (i.e., hidden layer width and depth) and hyperparameters (i.e., learning rate and weight decay parameter) were tuned via a random search minimizing mean-square-error (MSE) in conjunction with five-fold cross-validation using trials from the 12 subjects in the training set (Table I). To prevent exploding gradients, the identified learning rate was employed with a learning rate scheduler, which multiplied the learning rate by 0.75 every 100 epochs.

### D. Transfer Learning Model

We developed an ANN that was pre-trained on the simulated dataset and fine-tuned on the recorded dataset (Fig. 1, bottom). This transfer learning model maintained an LSTM architecture identical to the best-performing direct learning model to capture the complex mapping between recorded muscle activations and thumb-tip forces. However, the transfer learning model was trained from scratch using a different protocol, which involved additional hyperparameter tuning in the pre-training and fine-tuning steps. First, we used the simulated dataset to train the LSTM architecture to predict three-component thumb-tip forces from only muscle activations. During this pre-training step, we tuned the hyperparameters of the LSTM via a random search. Then, we froze the gradients of the hidden layers and concatenated the LSTM with the feedforward architecture. This transfer learning model was then fine-tuned with the recorded dataset, which included both dynamic muscle activations and 8 static features. The final model underwent a random search to identify the feedforward architecture and hyperparameters best suited to make predictions on the recorded data (based on MSE and in conjunction with five-fold cross-validation on trials from the 12 subjects in the training set), as well as the number of backend hidden layers to unfreeze in the LSTM, which was limited to 0, 1, or 2 (Table I). This model also employed a learning rate scheduler, which multiplied the learning rate by 0.75 every 100 epochs.

### E. Musculoskeletal Simulations for Comparison on the Leave-Out Set

Musculoskeletal simulations can be used to predict thumb-tip force without implementation of machine learning methods. Thus, to provide an additional basis of comparison for the predictive performance of the ANNs, we ran computed muscle control and forward dynamic musculoskeletal simulations for each subject in the leave-out set. Specifically, we anthropometrically scaled the generic wrist and thumb model to represent the 3 leave-out subjects. The musculoskeletal model contains 14 actuators, whereas we only recorded 8 muscle activations. Therefore, we used computed muscle control to simulate the activations of the remaining actuators, namely the *extensor carpi radialis longus* (ECRL), *extensor carpi radialis brevis* (ECRB), *extensor carpi ulnaris* (ECU), *flexor carpi radialis* (FCR), and *the abductor pollicis brevis* (APB). The oblique and transverse heads of the ADD are represented by two actuators, each of which were defined by activations equal to the recorded signal. The target force of each computed muscle control simulation matched that of the recorded thumb-tip force. The target posture for the computed muscle control simulations was static, and defined by −15° carpometacarpal flexion, −20° carpometacarpal abduction, 20° metacarpophalangeal flexion, and 40° interphalangeal flexion. The resulting simulated muscle activations for the ECRL, ECRB, ECU, FCR, and APB served as forward dynamic simulation inputs alongside the measured muscle activations for the remaining 8 muscles. To reduce noise, all recorded muscle activations were processed through a moving average filter before being input into forward dynamic simulations. Referencing the sum of absolute differences for each signal (effectively quantifying information altered) at varying window sizes (ranging 5 to 50 datapoints, in 5 datapoint increments), we selected a window size of 30 datapoints to balance noise reduction and signal preservation. Lastly, we evaluated the thumb-tip forces resulting from each forward dynamic simulation using joint reaction analysis.

### F. Validation & Analyses

To interpret the influence of transfer learning on ANN performance, we completed two evaluations: (1) we quantified validation errors by applying LOOCV to trials from the 12 subjects in the training set and (2) we quantified test errors on the final models using trials from the 3 leave-out subjects (test set). LOOCV enabled us to assess the consistency of each model’s performance across individual training subjects and to quantify average validation error. Test errors quantified each model’s generalizability to previously unseen subjects. We also quantified prediction errors associated with musculoskeletal simulations (representing each leave-out subject), providing additional context for each ANN’s predictive performance.

For both the LOOCV and final model evaluations, we evaluated each ANN’s predictions using various metrics. We quantified test root-mean-square errors (RMSEs) and mean-absolute errors (MAEs) for each the direct learning model and the transfer learning model. Errors were calculated between the predicted and the recorded thumb-tip forces. For the evaluation of final models, we also used a linear mixed-effects model (LME) to separately compare the residual and absolute errors; subjects were treated as a random effect, and significance was set at p < 0.05. Additionally, we visualized each final ANN’s predictions, plotting parity plots and each model’s predictions. These efforts enabled us to summarize the errors and visualize prediction bias. We displayed the standard deviation of each ANN’s predictions alongside those of the recorded data to elucidate the variability in each.

## III. Results

**Fig. 4.**
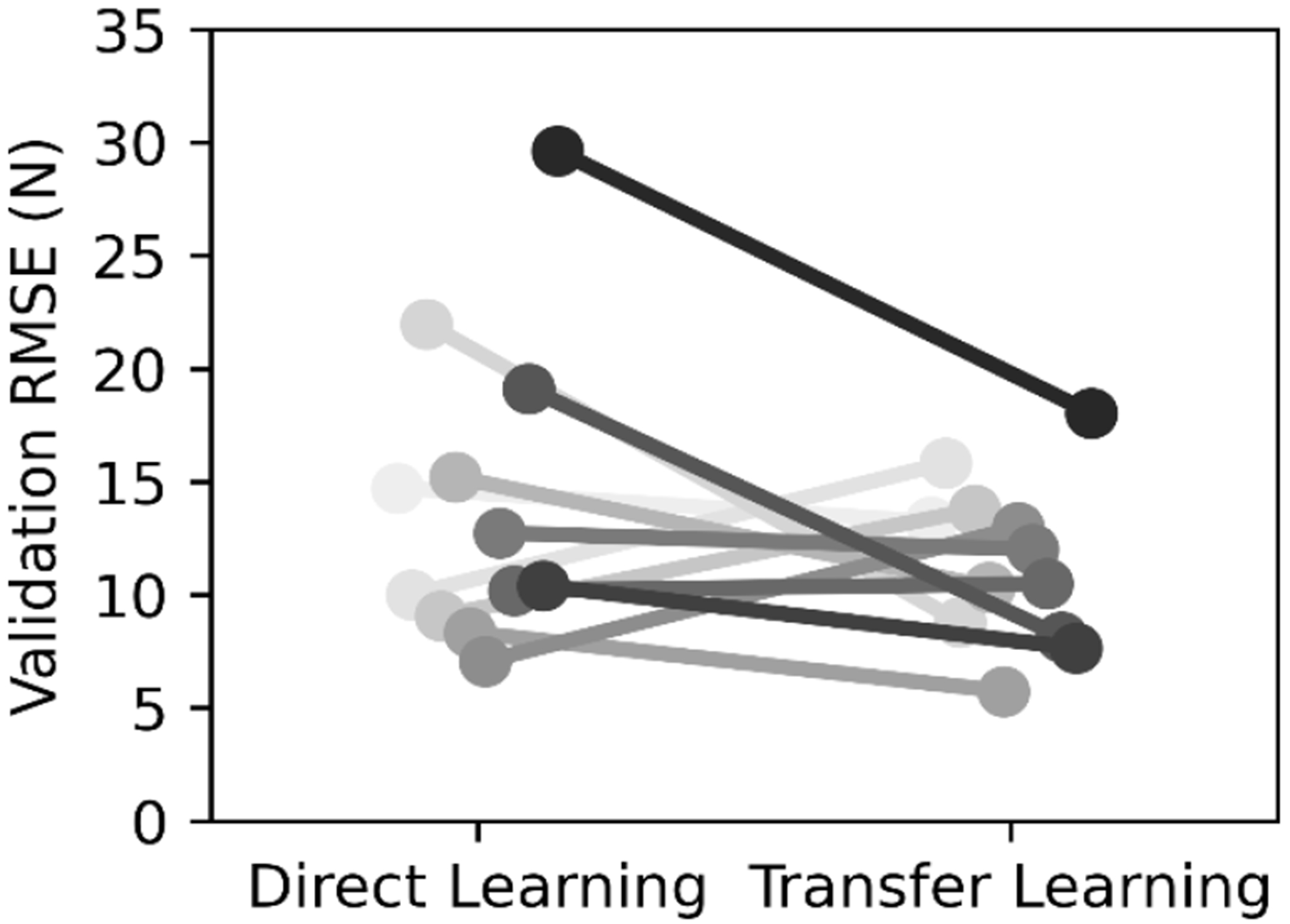
Validation RMSEs associated with LOOCV for the direct learning and transfer learning models. Each grayscale color corresponds to one of the 12 subjects in the training set.

Transfer learning consistently provided modest improvements to predictive performance relative to the direct learning model. Specifically, evaluations of the direct learning models from LOOCV yielded RMSEs of 14.0 ± 6.6 N and MAEs of 10.0 ± 4.5 N. By comparison, the transfer learning models yielded RMSEs of 11.4 ± 3.6 N and MAEs of 8.1 ± 2.5 N. A point plot of the validation errors per-subject illustrates that transfer learning models produced lower validation RMSEs than direct learning models for trials associated with 8 of the 12 subjects in the training set (Fig. 4).

**Fig. 5.**
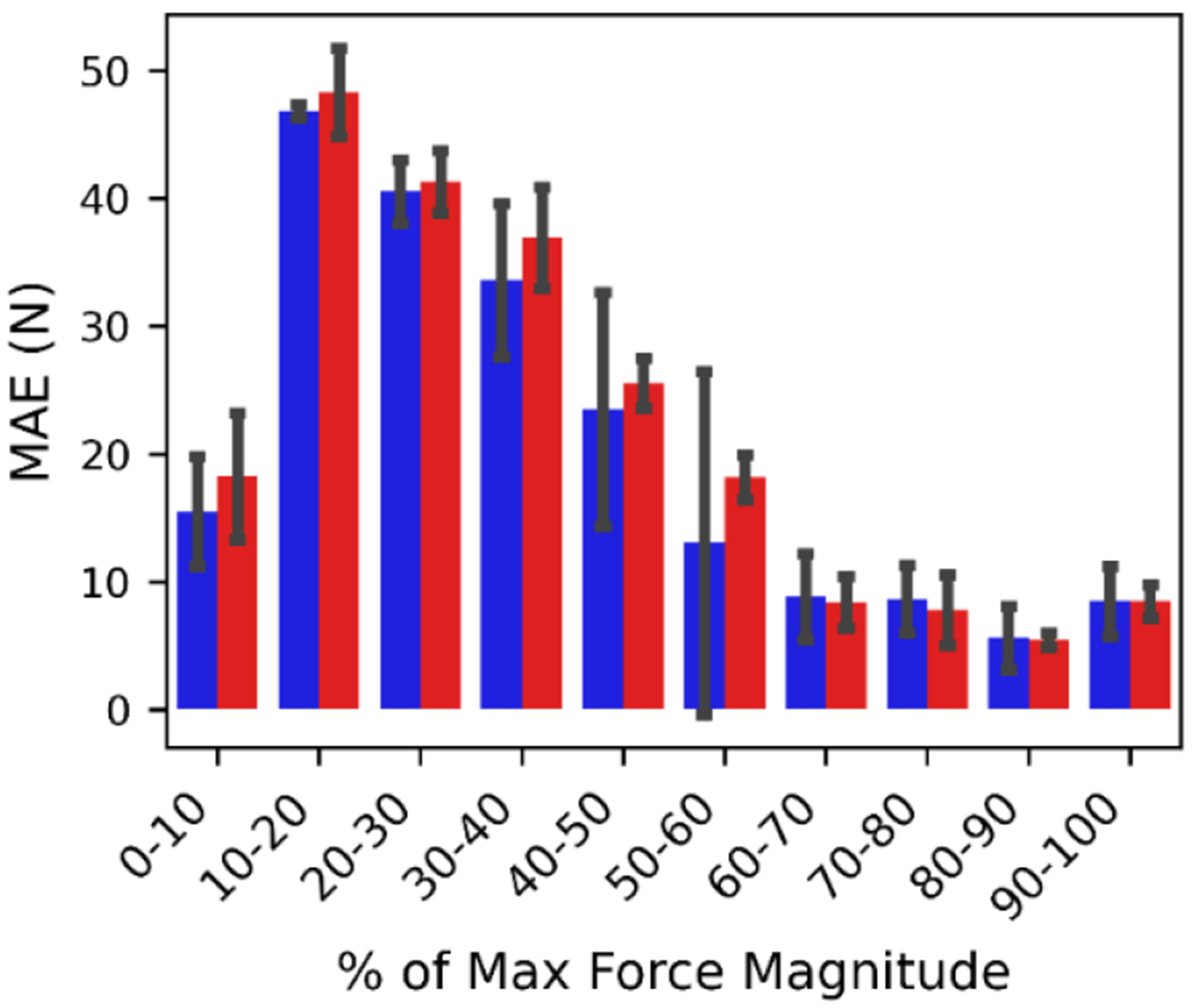
Test MAEs stratified by percentage of maximum force magnitude for the direct learning model (red) and transfer learning model (blue). Both models tended to decrease in prediction error with increasing force.

Test predictions of the final direct learning and transfer learning models yielded overall RMSEs of 12.1 N and 11.4 N, and MAEs of 7.2 N and 6.2 N, respectively. Across the three leave-out subjects, test RMSE ranged 11.6 N to 13.0 N for direct learning and 10.7 N to 12.7 N for transfer learning. Relative to the maximum recorded thumb-tip force magnitude in the leave-out set, these errors corresponded to approximately 11.7% and 11.0% (RMSE) and 7.0% and 6.0% (MAE) for the direct and transfer learning models, respectively. This indicates a decrease in error with the implementation of transfer learning of 5.8% and 13.9% for RMSE and MAE, respectively. Furthermore, when stratified by percentage of maximum force, RMSE tended to decrease as force magnitude increased (Fig. 5).

Both the direct learning and transfer learning models substantially outperformed the musculoskeletal simulations for each leave-out subject, which yielded an RMSE of 22.9 N and MAE of 17.9 N, corresponding to approximately 22.2% and 17.3% of maximum thumb-tip force magnitude. For context, the recorded thumb-tip forces had a mean magnitude of 59.8 N with a standard deviation of 30.3 N (summarizing across all 15 subjects and all trials).

**Fig. 6.**
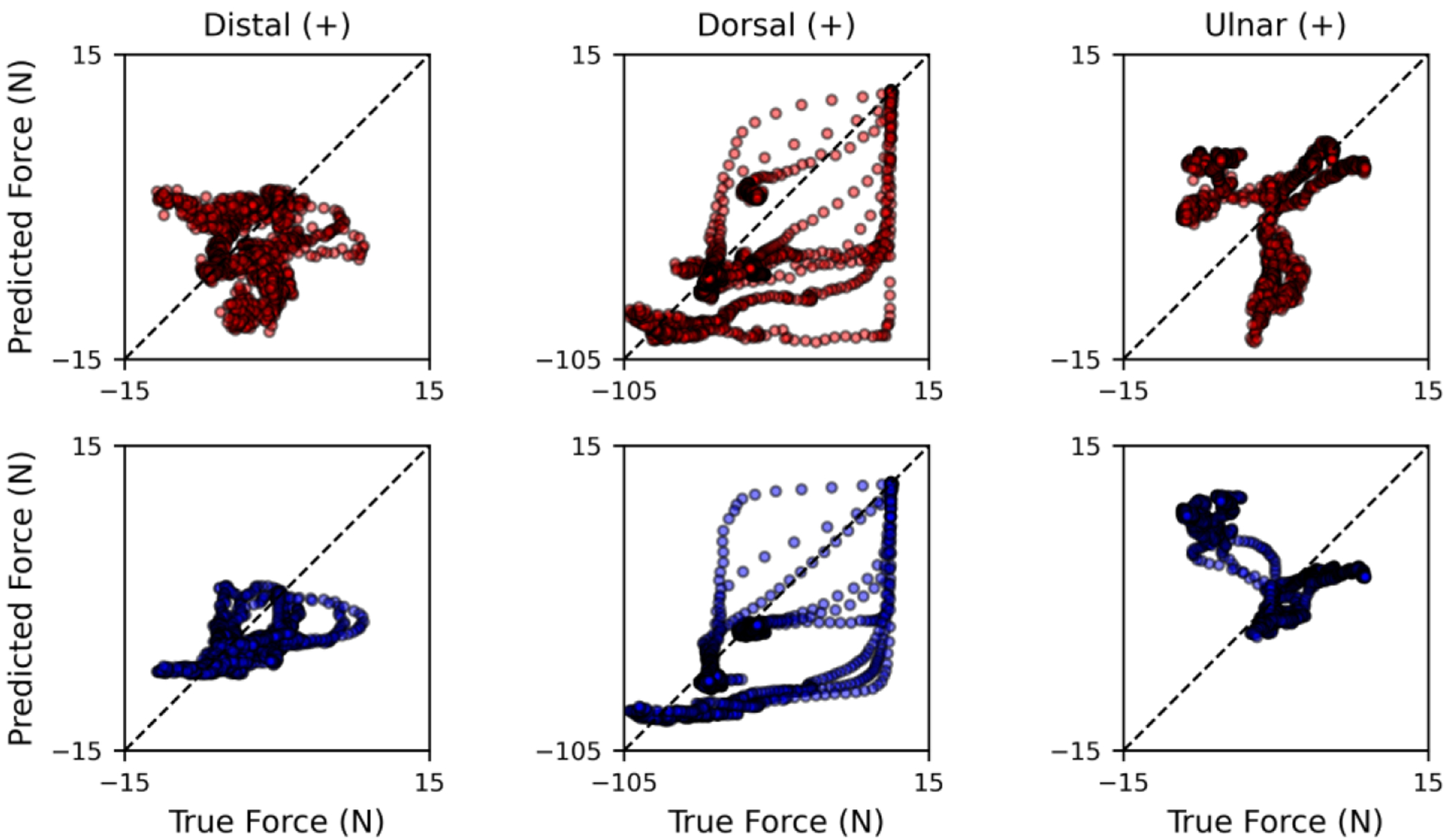
Parity plots of three-component thumb-tip forces as predicted by the direct learning model (red) and transfer learning model (blue). Each plot displays predictions for 9 trials (3 leave-out subjects performing 3 repetitions each). Both models produced the greatest prediction errors during the ramp to maximal force, as shown by the high distances between points and the parity line for low true thumb-tip forces (e.g., predictions for distal thumb-tip component).

Although the residual errors were not significantly different between the transfer learning model and direct learning models (p = 0.105), the absolute errors were significantly lower in the transfer learning model (p = 0.04). Parity plots for the leave-out set predictions (Fig. 6) reveal that both ANNs generalized better to the distal and dorsal force components than to the ulnar component. The transfer learning model predicted distal and dorsal thumb-tip forces with lower errors than the direct learning model (Table II).

**Fig. 7.**
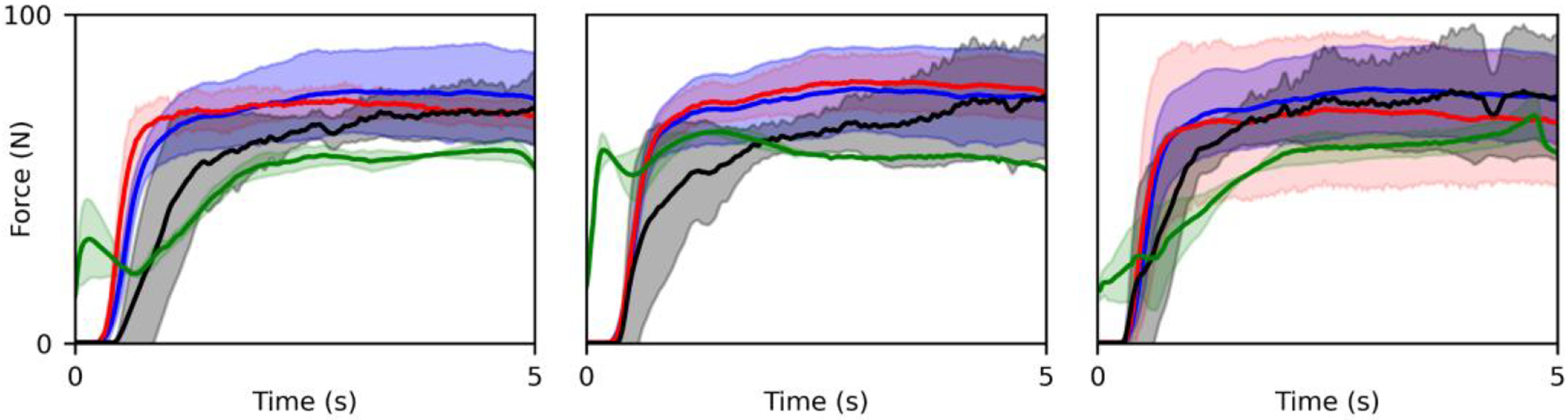
Thumb-tip force magnitudes as recorded (black) and predicted by the direct learning model (red), transfer learning model (blue), and forward dynamic simulations using scaled musculoskeletal models (green). Each subplot corresponds to one leave-out subject. Shaded regions represent standard deviations across each of 3 repetitions of lateral pinch. While both machine learning models regressed high-magnitude thumb-tip forces with low error, the variability of the direct learning model’s predictions was not consistent across subjects.

The transfer learning model performed more consistently across each leave-out subject, as evidenced by plots of the magnitudes of each model’s predictions (Fig. 7). For example, the right-most subplot highlights that the direct learning model’s predictions for one subject display a markedly high standard deviation relative to its estimations for the other two subjects. Furthermore, both the direct and transfer learning models produced lower errors at high-magnitude thumb-tip forces than during the ramp up to maximal force. Overall, the standard deviations of the predictions for the direct learning and transfer learning models, 11.3 N and 10.8 N, respectively, were similar to those of the recorded data.

## IV. Discussion

The transfer learning model, equipped with knowledge of musculoskeletal simulations and recorded data, outperformed the direct learning model when evaluated on a leave-out set. Compared to the direct learning model, the transfer learning model showed lower RMSE and MAE, and a statistically significant decrease in absolute error across all subjects and trials (p< 0.05). While the reductions in RMSE and MAE were modest, their consistency across subjects suggests that transfer learning offers a practically meaningful improvement to machine learning model development. Although residual errors were lower in the transfer learning model, this effect was not significant. These findings suggest that transfer learning reduced the magnitude of the prediction errors without substantially altering directional bias. Furthermore, prediction errors were axis-dependent (Table II), with transfer learning outperforming direct learning in the distal and dorsal directions. In contrast, performance in the ulnar component was not improved with transfer learning, which may reflect differences in how this force component is represented in the simulations or its comparatively smaller contribution to lateral pinch. Parity plots also revealed axis-dependent deviations in forces, displaying a subtle tendency for the transfer learning model to overpredict ulnar forces (Fig. 6). Overall, our results indicate that the transfer learning model’s predictions tended to deviate less from the recorded force values. Even though both machine learning models outperformed musculoskeletal simulations, the prediction errors should also be interpreted in the context of functional demands. Many lateral pinch tasks require forces on the order of 10 N, with tasks such as using a remote, zipper, or key requiring less than 5 N of pinch force [32]. Given this information, the modest improvements in MAE observed with transfer learning approach a functionally meaningful scale. Nonetheless, further model refinement may be necessary for many real-world applications. Even though both the direct learning model and transfer learning model outperformed musculoskeletal simulations, our results suggest that simulations encode information valuable for the prediction of lateral pinch thumb-tip forces. Thus, the developed transfer learning framework is a viable approach to enhance predictions of upper extremity biomechanics.

Multiple studies have advocated for the use of transfer learning to improve machine learning model performance when few training observations are available (e.g., [17], [33]). Our results support these prior works by demonstrating that errors associated with predicting three-component thumb-tip forces decreased with the use of transfer learning when examining a biomechanical task that is challenging and time-intensive to study experimentally. Recording a complete dataset of forearm and hand muscle activations is cumbersome, as these signals require the use of invasive fine-wire electrodes, and some subjects may elect not to have signals from fine-wire electrodes recorded due to discomfort during needle insertion. For example, only 15 of the recruited 29 subjects for our study completed all insertions. Furthermore, collecting biomechanical data costs time both for the researchers and human subjects involved. In this study, recording upper extremity muscle activations and forces during muscle-specific MVCs and lateral pinch for one subject (who completed all insertions) required approximately 2-3 hours of data collection (prior to any data processing). By comparison, simulating one subject’s muscle activations during lateral pinch requires approximately 4.22 core-minutes [34]. By pre-training on simulated data, our transfer learning framework can bolster models that rely on muscle activations from the forearm and hand.

Toward our goal of improving models of the upper extremity, we employed a novel ANN architecture uncommon to biomechanics literature. LSTMs can detect patterns in dynamic data, such as the muscle activations we recorded. Therefore, these architectures are widely applied when predicting time-series biomechanical features, such as joint angles and forces [35], [36]. However, few works take the additional step of concatenating other ANN architectures together, such as to enable learning with multimodal data. By concatenating the LSTM with a feedforward architecture, the force predictions of our ANNs were informed both by rapidly changing muscle activations and easily-measurable static subject-specific features. Future works may adopt this practice to improve the generalizability of other models, perhaps investigating the features that best describe biomechanical attributes across populations.

Discrepancies between simulated and recorded datasets likely influenced the efficacy of our transfer learning framework. Notably, simulation-to-real transfer learning involves freezing ANN layers pre-trained on simulation data before fine-tuning on recorded data. To enhance predictive performance, the frozen layers should encode physically meaningful representations learned from the simulations. To benefit generalizability through transfer learning, those representations should, at least in-part, also be present in the recorded dataset. However, there were multiple discrepancies between our simulated dataset and recorded dataset. For example, the musculoskeletal simulations we employed for pre-training and validation were scaled anthropometrically but were not otherwise optimized for the young adult population captured in our recorded dataset. Furthermore, temporal discrepancies, such as differences in sampling rate, frequency content, and force-generation timing (e.g., prescribed simulation ramps versus subject-driven rapid force initiation), as well as unmodeled neuromechanical factors such as co-contraction variability and EMG measurement noise, may affect how muscle activation dynamics are represented and mapped to thumb-tip forces. Future efforts should aim to mitigate these differences and thereby potentially improve the effectiveness of simulation-to-real data transfer learning for capturing biomechanics.

Recording biomechanical data of the upper extremity (especially muscle activations) remains challenging. Thus, the present work was limited in the number of recorded observations with which we could train and test each ANN. While transfer learning mitigates the need for large biomechanical datasets, future work should consider dataset size in comparison to the desired model’s complexity. Furthermore, the present work validated a transfer learning framework by only modeling lateral pinch. Our framework is adaptable and presently employed to map muscle activations and easily-recorded static subject-specific features to three-component forces. However, the transferability of our findings to other biomechanical tasks warrants further investigation.

Our results demonstrate that simulation-to-real data transfer learning can provide improvements to the prediction of lateral pinch forces, even when using a relatively small, recorded dataset. We specifically demonstrated that an ANN pre-trained on simulated data and fine-tuned on recorded data can predict dynamic three-component thumb-tip forces from muscle activations and static subject-specific features. Our results indicate that musculoskeletal simulations contain knowledge valuable to the prediction of upper extremity forces. Thus, as the fields of computational and experimental biomechanics progress, musculoskeletal simulations will likely emerge as an important tool for pre-training robust biomechanical machine learning models. Such advances may facilitate the development of robust data-driven tools across a range of applications, improving predictive performance when large, recorded datasets are scarce.

## Figures and Tables

**TABLE I:** Architecture and Hyperparameters of the Direct Learning and Transfer Learning Models

	Direct Learning	Transfer Learning
Learning Rate	8.2 × 10^−3^	8.3 × 10^−3^
Weight Decay	6.0 × 10^−2^	9.2 × 10^−2^
LSTM Hidden Layers	8	8
LSTM Hidden Layer Width	18	18
Backend LSTM	N/A	2
Layers Unfrozen		
Feedforward Hidden Layers	1	2
Feedforward Hidden Layer Width	14	26

**TABLE II:** Prediction Errors Across Thumb-Tip Force Components Associated with Direct and Transfer Learning Models

	Distal	Dorsal	Ulnar	Metric^1^
Direct Learning	4.5	19.4	6.5	RMSE
Transfer Learning	3.0	17.7	8.3	

Direct Learning	3.5	13.0	5.2	MAE
Transfer Learning	2.3	10.8	5.7	

1 Errors shown correspond to predictions on a leave-out set, in Newtons.
